# FAMILY CAREGIVING FOR OLDER ADULTS IN LONG-TERM CARE DURING THE PANDEMIC: STRESS, SOCIAL SUPPORT, AND ADAPTATION

**DOI:** 10.1093/geroni/igac059.107

**Published:** 2022-12-20

**Authors:** Geunhye Park, Erin Robinson, Gashaye Melaku Tefera

**Affiliations:** University of Missouri, Columbia, Missouri, United States; University of Missouri, Columbia, Missouri, United States; University of Missouri, Columbia, Missouri, United States

## Abstract

Older adults residing in long-term care (LTC) are especially vulnerable to the COVID-19 pandemic. Federal and local health officials have issued strict visitation guidelines, including family caregivers. Given that family caregivers are essential in the well-being for older adults in LTC, these measures have had an enormous impact. As little is known about the experiences of family caregivers, the purpose of this study was to explore how the COVID-19 pandemic impacted family caregivers’ roles, mental health, and adaptation. Semi-structured interviews (N=25) were conducted with family caregivers of older adults in LTC (Mean age= 59.7; 92% female) via phone/Zoom. An interview guide led the question asking process and participants were asked open-ended questions about the impact of COVID-19 related protocols on their caregiving, mental health, and sources of social support. Interviews were transcribed verbatim and analyzed in Nvivo, guided by Grounded Theory methods. The majority of participants (76%) identified as a child of their care recipient. Findings highlight that most participants experienced numerous changes to their caregiving tasks, such as assisting with activities of daily living (ADLs), limited monitoring for their loved ones, and a reduction of social support provided to the care recipient. Family caregivers also reported other changes in their roles that resulted in increased stress and mental health concerns. These concerns were discussed in detail, including ways in which family caregivers adapted to their new roles and managed their stress. Findings from this study will inform interventions geared to better support family caregivers, particularly during times of crisis.

